# Restoration of clipped seismic waveforms using projection onto convex sets method

**DOI:** 10.1038/srep39056

**Published:** 2016-12-14

**Authors:** Jinhai Zhang, Jinlai Hao, Xu Zhao, Shuqin Wang, Lianfeng Zhao, Weimin Wang, Zhenxing Yao

**Affiliations:** 1 Key Laboratory of Earth and Planetary Physics, Institute of Geology and Geophysics, Chinese Academy of Sciences, Beijing 100029, China; 2 School of Information Engineering, Minzu University of China, Beijing 100081, China; 3 Key Laboratory of Continental Collision and Plateau Uplift, Institute of Tibetan Plateau Research, Chinese Academy of Sciences, Beijing 100101, China

## Abstract

The seismic waveforms would be clipped when the amplitude exceeds the upper-limit dynamic range of seismometer. Clipped waveforms are typically assumed not useful and seldom used in waveform-based research. Here, we assume the clipped components of the waveform share the same frequency content with the un-clipped components. We leverage this similarity to convert clipped waveforms to true waveforms by iteratively reconstructing the frequency spectrum using the projection onto convex sets method. Using artificially clipped data we find that statistically the restoration error is ~1% and ~5% when clipped at 70% and 40% peak amplitude, respectively. We verify our method using real data recorded at co-located seismometers that have different gain controls, one set to record large amplitudes on scale and the other set to record low amplitudes on scale. Using our restoration method we recover 87 out of 93 clipped broadband records from the 2013 Mw6.6 Lushan earthquake. Estimating that we recover 20 clipped waveforms for each M5.0+ earthquake, so for the ~1,500 M5.0+ events that occur each year we could restore ~30,000 clipped waveforms each year, which would greatly enhance useable waveform data archives. These restored waveform data would also improve the azimuthal station coverage and spatial footprint.

Seismic waves can penetrate deep into the Earth providing key information to help us detect geometric structures and physical characteristics of the Earth’s interior^1^,^2^. Characteristics of a seismic waveform can help us understand regional structures within our Earth and even the dynamic history of the Earth^3^,^4^,^5^. Our understanding of high-resolution spatial complexities and deep Earth structures is limited, however, by the number of seismic stations and the quality of the data they recorded. One fundamental solution is to increase the density of evenly distributed seismic stations (either permanent or temporary, on land or ocean bottom). But increasing station density is problematic because of the high costs of instrument deployment and maintenance^6^,^7^,^8^. So instead, we ask the question how we can use existing data from the ~6,000 seismic stations (http://ds.iris.edu/wilber3/find_event) currently deployed around the world to increase useful seismic waveform archives?

Most broadband seismic stations need a large enough gain control to detect fairly weak signals from teleseismic events. With this setting, however, a large local or regional earthquake would generate seismic wave amplitudes that are too strong to be recorded on-scale by the broadband seismometers. In other words, the amplitude would be saturated (e.g. exhibiting as trapezoid) if the amplitude reaches the upper-limit dynamic range of the seismometers^9^,^10^,^11^. Amplitude-clipped data are typically assumed to be worthless and are seldom used in waveform-based applications (e.g., full waveform inversion, focal mechanisms, and receiver function). For a clipped waveform even basic processing (e.g. bandpass filtering, and removing instrument response) is problematic because of the aliasing of the frequency components (also called frequency leakage)^12^, since most basic processing procedures involve convolution or deconvolution that are basically frequency-based operations. To perform these basic processing procedures, one could select only the unclipped portions of the waveform, but this would require applying a damping taper function on the unclipped portions of the waveform that are close to the clipped portions, which causes additional waste of seismic data (usually 30 to 50 samples).

The seismic records close to the epicenter convey much more valuable information of the earthquake source and the regional structures than data from stations further away. By definition typically waveform amplitudes are larger closer to the earthquake epicenter and therefore more prone to be clipped. With the increasing density of seismic stations (especially for temporary seismic array), we anticipate that the total number of clipped waveforms is only going to increase in the future. Therefore, it is imperative for us to find a way to productively use clipped data.

There have been previous studies that investigate how to use clipped data. Galbraith & MacMinn^9^ use a two-sided interpolation operator derived by least-squares to accomplish the task. Karabulut & Bouchon^10^ recover the clipped part of the waveform using cubic spline interpolation. In the presence of many consecutive clipped samples these two methods would encounter large errors and not be feasible. Yang & Ben-Zion^11^ consider waveform corrections based on a linear interpolation applying the Kriging method and using similar unclipped waveforms from nearby smaller events. The Kriging method is shown to perform well in the presence of few consecutive clipped samples. In cases with six or more consecutive clipped samples, the similar waveform method generally performs better, especially if there is a high cross-correlation coefficient between the clipped waveform and the reference waveform. However, corrections using similar waveforms are likely to produce overly large errors when the cross-correlation coefficient is small. In fact, it is sometimes difficult to obtain similar waveforms, especially near new faults or where there is no previous record available.

Here, we assume that the seismic data are band limited and the clipped parts of the waveform share the same frequency content as the un-clipped parts. Using these two basic assumptions, we can use digital image processing and seismic exploration field methods to repair clipped waveforms. We reconstruct (or restore) clipped records using an advanced signal-processing technique: the projection onto convex sets (POCS) method^13^,^14^,^15^, which is popular in studies of restoration of images or seismic data that have inadequate spatial sampling. To the best of our knowledge, this work is the first to restore consecutively clipped data using the POCS method. Theoretical analyses and numerical experiments show that the POCS method can substantially improve the availability of useful near-field data recordings.

## Theory

### Waveform restoration using POCS method

**Figure 1:**
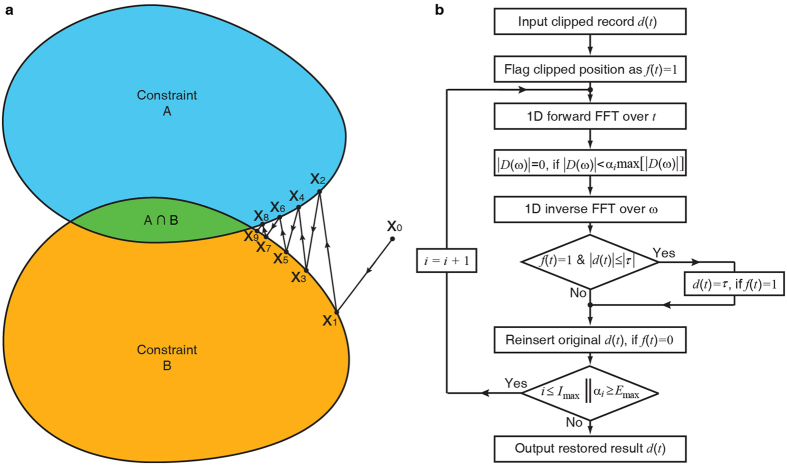
The basic idea of the POCS method (**a**) and the flow chart of restoring a clipped seismic record using the POCS method (**b**). The fast Fourier transform is abbreviated as FFT, and the inverse FFT is abbreviated as IFFT. *D(ω*) = *FFT*[*d(t*)]. The threshold *τ* is the clipped level, as shown in equation (1). For simplicity, we assume that max[*s(t*)] = 1 in this flowchart. We flag the clipped positions as *f(t*) = 1 and the non-clipped positions as *f(t*) = 0. The maximum iteration times are defined as *I*_max_, and the error tolerance is defined as *E*_max_.

Data gaps occur when samples are missing when the detector fails or could not be installed at the sampling positions designated. Image processing techniques and methods used in seismic exploration have developed many powerful tools to restore missing samples in gappy data^12^,^14^,^16^. In this paper, we regard the clipped waveform as an imperfectly sampled signal, to which we can apply these powerful tools. Our goal is to restore the large amplitude samples that have been truncated, also known as ‘clipped’. One thing that differs between our data and those data used in many image processing techniques is that in our work the clipped data only locate around peak amplitudes, whereas in traditional image processing missing samples are not limited to a specific time or amplitude range. After investigating a number of different methodologies, we select the POCS method^13^,^14^,^15^,^17^,^18^,^19^ because its physical meaning is clear and its algorithm is robust. The basic idea of the POCS method is shown in Fig. 1a. We are able to obtain a restored waveform by gradually imposing *a priori* constraints on the waveform output from different aspects. An optimal solution can be iteratively obtained by shuttling between various constrains of different aspects of the waveform, since the optimal solution should belong to the intersection of all possible convex sets (or constrains).

If some samples are missing in an evenly sampled record, its frequency spectrum would suffer from frequency leakage^12^,^14^. In other words, the peak amplitude of the frequency spectrum would be slightly smaller than that of the perfect signals, and these missing energies would be superposed at the wrong frequencies. By properly rearranging most of the energy, we can repair the frequency leakage, and the original signal can be restored. Iteratively, the POCS method can gradually extract reliable frequencies from the polluted frequency spectrum, since large-energy frequency components usually have smaller pollutions from the other frequencies even though their amplitudes are not correct.

**Figure 2:**
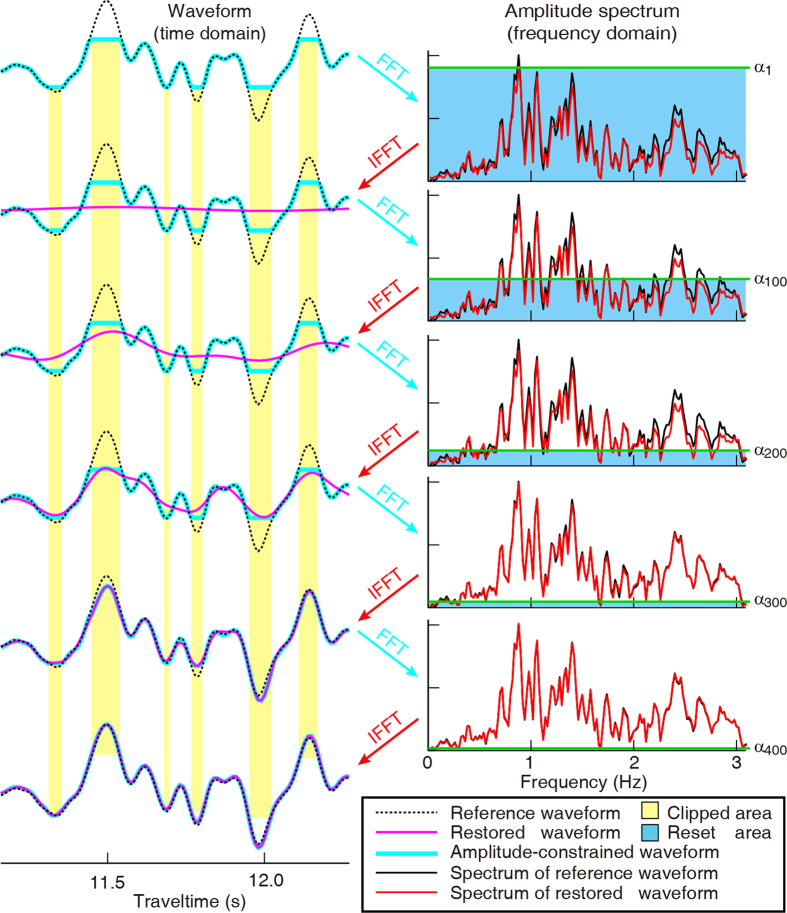
Illustration of how we use the POCS method to restore a clipped seismic record. Of the ~500 iterations preformed, here we show only every ~100 iterations.

Figure 1b shows the flowchart of the POCS method for restoring the clipped waveforms. We define the waveforms in the time domain as set A and the frequency spectrum as set B, respectively. The set B is evolving under a gradually decreasing threshold to reduce the frequency leakage caused by the missing samples (actually clipped). Our method includes the following process: (1) We flag clipped samples with a 1 marker and non-clipped samples with a 0 marker. (2) We perform a forward Fourier transform on the data. (3) We leave reliable frequency components untouched if their amplitudes are over a given threshold, then, we perform an inverse Fourier transform over these reliable big-amplitude components. (4) We reinsert those unclipped samples in time domain and assign any amplitude that is smaller than the clipped threshold level at clipped positions to be equal to the clipped level. And (5) we slightly decrease the threshold^14^,^19^ and repeat the above procedure until the results stabilize. Using this method we find that both the waveform and the spectrum approach the true theoretical solution gradually after ~300 iterations, as shown in Fig. 2. A linear threshold is associated with a slow convergence but the results would be more robust compared with an exponential threshold, as shown in digital image processing by Wang and Zhang^19^.

### Verifying the POCS method using intentionally clipped synthetic waveforms

We verify the feasibility of the POCS method by devising a numerical experiment using a base set of robust unclipped seismic waveform. We first intentionally clip the peak amplitudes of the waveforms using a series of different clipped levels (10~90%) shown in equation (1); then, we restore each of the clipped waveforms using the POCS method; finally, we compare the final results with the unclipped waveform. In this way we can evaluate the performance of the POCS method for different clipped levels.

**Figure 3:**
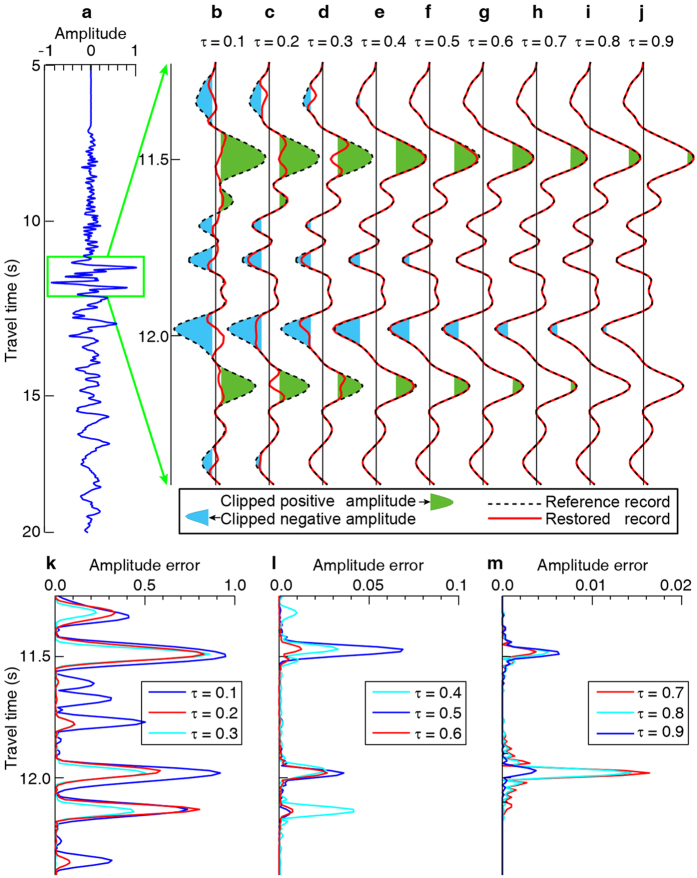
Different clipped levels and corresponding waveform restoration results. (**a**) True unclipped seismic record, which we use as a reference for checking the accuracy. (**b**–**j**) Application of different clipped levels (*τ*, range 0.1–0.9) with respect to the maximum waveform amplitude. The clipped parts of the waveforms are depicted with filled cyan and green patches of colors. (**k**–**m**) relative errors for waveforms clipped at different levels. For an explanation of the clipped level *τ* see equation (1).

We define the clipped level as a proportion to the peak amplitude of the unclipped record as follows


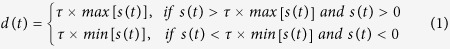


where *d(t*) is our synthetic clipped record that we create by resampling a perfect seismic signal *s(t*) using a threshold (i.e., the clipped level) of *τ*. We use a unified threshold for the simplicity of illustrating the validity of the POCS method using synthetic data (Fig. 3). When processing real data, we typically use different thresholds for the positive and negative peaks of the waveform. We defined the weak (τ >= 0.7), moderate (0.4 < =  τ< 0.7) and strong (τ < 0.4) according to the clipped level relative to the maximum amplitude. When we do not know the exact maximum amplitude (this is the case for practical applications), we can estimate the clipped level roughly by the total number of consecutive clipped samples. For a sampling interval of 0.01 s, a total number of consecutive clipped samples of 1~25, 26~50, and >50 generally corresponds to “weak”, “moderate”, and “strong”, respectively. For a given number of consecutive clipped samples, the clipped level would be higher for a high frequency component compared with a low frequency component, since the clipped level is dependent on the frequency.

As shown in Fig. 3, when the clipped level is at 70~90% of the peak amplitude (i.e., τ = 0.7~0.9), the restoration error is only 0.7~1.7% of the peak amplitude (Fig. 3k). When the clipped level is more at 40~60% (i.e., τ = 0.4~0.6), the error increases to 3~7% (Fig. 3l). At clipped levels of 10~30% (i.e., τ = 0.1~0.3), we have much larger errors of 70~90% (Fig. 3m). These tests indicate that the POCS method can successfully restore the clipped waveforms when the clipped level is weak and when the clipped level is moderate, however the POCS method is not able to restore waveforms that are strongly clipped (i.e., when more than two-third’s of the peak amplitude is clipped).

## Results

### Waveform restoration for far- and near-field seismograms

**Figure 4:**
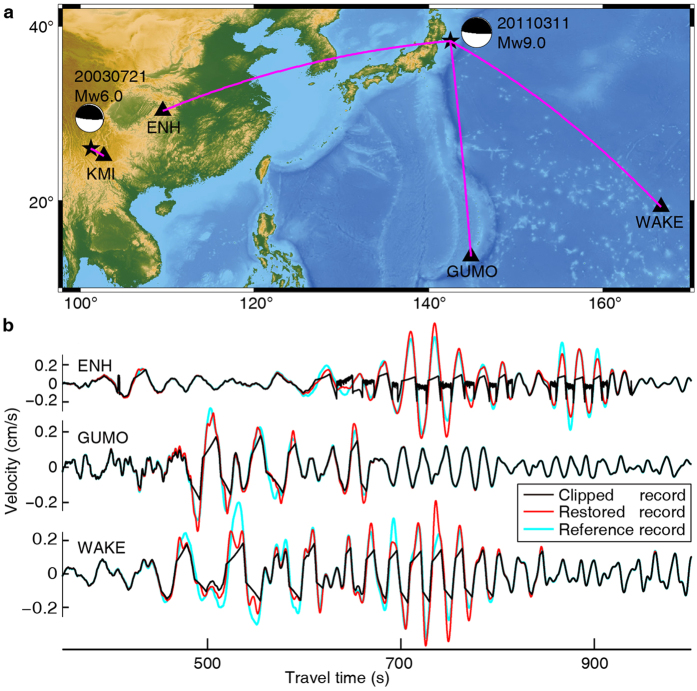
Waveform restoration of seismograms recorded in the far-field. (**a**) Study region map that includes the mainshock Epicenter (star and associated focal mechanism) and select stations (black triangles) that recorded the 2011 Mw 9.0 Tohuko earthquake; (**b**) Waveforms: clipped (black curves), restored (red curves), and reference (cyan curves). The clipped and reference records are recorded by two co-located seismometers that have different gain controls. This figure was generated using the Generic Mapping Tools^20^ version 4.5.8 (http://gmt.soest.hawaii.edu).

**Figure 5:**
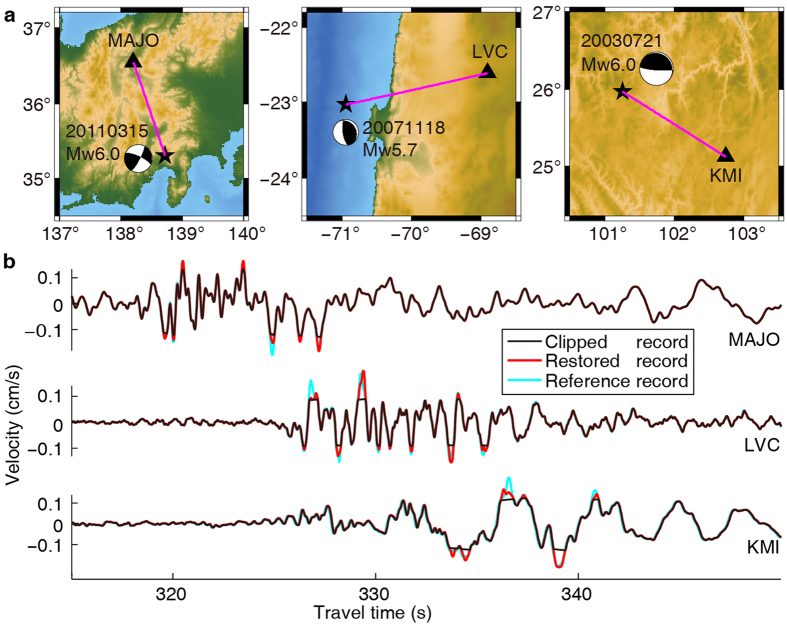
Waveform restoration of seismograms recorded in the near-field. (**a**) Epicenter (star and associated focal mechanism) and select stations (black triangles) for three regional earthquakes. (**b**) Waveforms: clipped (black curves), restored (red curves), and reference (cyan curves). The clipped and reference records are recorded by two co-located seismometers that have different gain controls. The right panel of (**a**) is also shown in the left side of Fig. 4a. This figure was generated using the Generic Mapping Tools^26^ version 4.5.8 (http://gmt.soest.hawaii.edu).

To verify that our method works reliably on real data, we next test our waveform restoration method using seismograms recorded in the far- and near-fields (Figs 4 and 5). The epicenteral distance is about 2000~3000 km for the far-field recordings and 200~300 km for the near-field recordings. We select data from three permanent stations that each has two co-located seismometers installed that have different gain controls. We regard the unclipped waveform from the small gain control station as the reference. We find that the POCS method can restore the clipped waveforms (Figs 4b and 5b), although some of the large amplitude peaks still have some errors. Importantly, these restored waveforms do not change the unclipped parts of the waveforms and the restored parts are seamless to those unclipped parts. This work shows that the POCS waveform restoration process is successful in restoring waveform data from far- and near-field recordings that contain weakly and moderately clipped seismic data, and can provide additional data that would otherwise not exist.

### Waveform restoration to the 2013 Mw 6.6 Lushan earthquake

**Figure 6:**
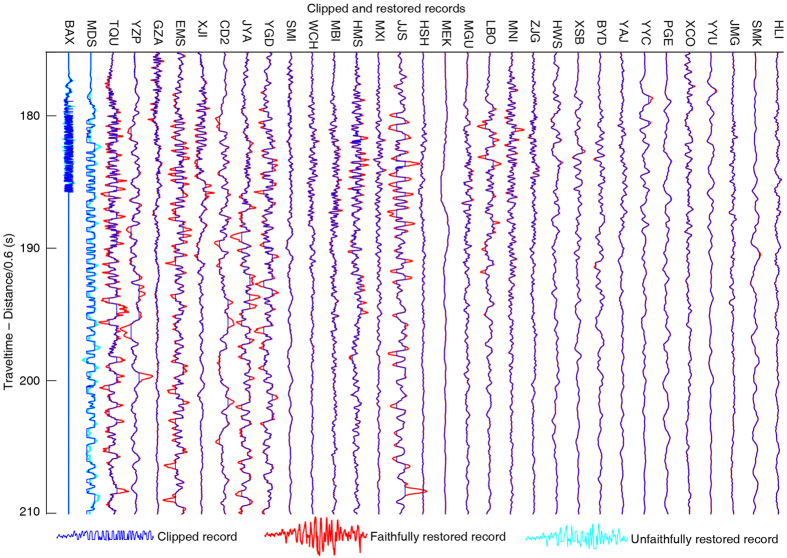
Depiction of clipped and restored broadband WE-component records of the 2013 Mw 6.6 Lushan earthquake. Comparison between the clipped (blue) and restored (red) records for the time period 177-210. The total number of clipped WE-component records is 33, of which we are able to restored 31 (Table 2). Records that we are not able to restore are shown in cyan. Stations are ordered by epicenteral distance. The records are aligned to show all waveforms within a small time window.

**Figure 7:**
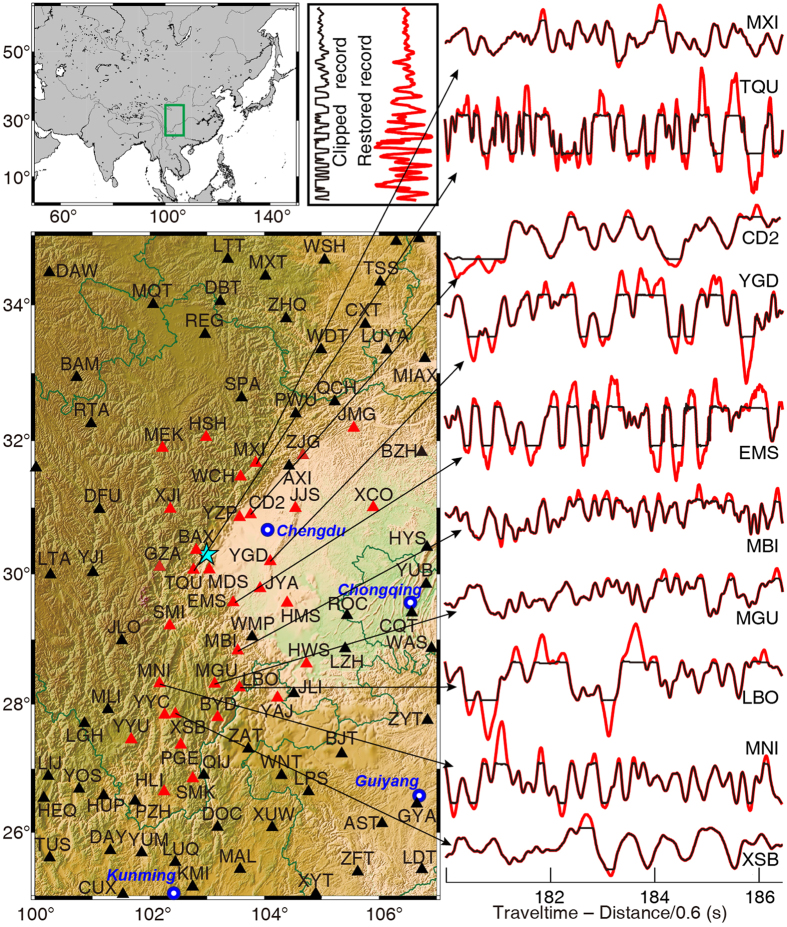
Waveform restoration results of the 2013 Mw 6.6 Lushan earthquake (aqua star) data. These records show WE-component broadband waveforms recorded by the regional Sichuan Seismic Network^21^. The clipped waveform data (black) are superposed on the restored waveforms (red). Red triangles denote the location of 33 stations that recorded clipped data. Figure 6 and Supplementary Figs 1–2 and Table 1 give all station information and waveform restoration results. This figure was generated using the Generic Mapping Tools^26^ version 4.5.8 (http://gmt.soest.hawaii.edu).

As a final test we apply our waveform restoration method to the 2013 Mw 6.6 Lushan earthquake to recover the clipped waveforms that occur primarily in the near-field^21^. We estimate that at least 38 of the 3-component broadband seismic station recordings contain clipped waveforms (Table 1). Using our method we are able to restore 88 out of 93 clipped waveforms (Table 2 and Fig. 6, Supplementary Figs 1–2). The total number of clipped waveforms is 33, 31 and 29 in west-east (WE), north-south (NS), and up-down (Z) components, respectively. Taking the WE component as an example (Figs 6 and 7), the unclipped parts of the waveforms remain unchanged in the restored waveforms. The two stations (BAX and MDS) close to the epicenter (~20 km) are too severely clipped to be restored using the POCS method. The other 31 stations generally show successfully restored results, especially for the moderately and weakly clipped records. The recording closest to the epicenter that we can successfully restore is the station TQU, which has an epicenteral distance of ~32 km. The furthest stations (JMG and HLI) that recorded clipped waveforms are ~320 to 370 km away from the epicenter.

### An estimation of the total number of clipped waveforms that can be restored

We next estimate the potential of the POCS method in restoring clipped waveforms within the China region. Here, we focus only on waveforms that we expect are weakly or moderately clipped (see definitions above) that can be adequately restored. We select events with magnitudes of 5.0 ≤ Ms ≤ 6.5 in the International Seismological Centre catalog (http://www.isc.ac.uk) that occurred between 1990-01-01 00:00:00 and 2013-03-29 00:00:00. Our study area includes China and adjacent areas (latitude 15°–55° and longitude 72°–135°). Given these constraints, we net 909 events. If we were to extend the spatial region to include the entire world, while keeping the same time and magnitude restrictions, the total number of events would be 11,970 (i.e., here we are investigating <10% of what might be possible). Taking the Mw 6.6 Lushan earthquake as an example, the total number of real-time seismic stations that recorded this mainshock is 1013 stations from the China Earthquake Networks (not including temporary stations and China Array) and is 1907 stations listed in the IRIS data catalog (http://ds.iris.edu/wilber3/find_stations/4215680).

Assuming that 60 waveforms (i.e., 3 components from our target 20 stations) are clipped during a large earthquake (5.0 ≤ Ms ≤ 6.5), we estimate the number of waveforms that can be adequately restored (say 50 out of 60) would reach 11,970 × 50 = 598, 500 ≈ 600,000 waveforms. If we have only 20 clipped waveforms (~8 stations involved) for each earthquake, we can still produce 11,970 × 20 = 239, 400 ≈ 230,000 restored waveforms. Note that these estimates are only for a maximum magnitude of 6.5, and our time window is limited to 1990 and 2013. In addition, the waveforms recorded by the widely used short-period seismometers are much easier to be clipped, and the total number of clipped waveforms would be fairly large but not easy to be estimated. Taking all these factors into account, the total number of clipped waveforms that we can recover would reach ~300,000, conservatively. Estimating that we are able to recover 20 clipped waveforms for each M5.0+ earthquake, so for the ~1,500 M5.0+ events that occur each year we could restore ~30,000 clipped waveforms each year, which would greatly enhance useable waveform data archives. In the future when we expect the station density to be much higher, especially for aftershock temporary seismic array deployments, there would be many more clipped waveforms.

## Discussion and Conclusions

There are three main advantages of using the POCS technique to restore clipped waveforms, First, the only assumption required is that the clipped seismic data are band limited and the clipped parts share the same frequency components with the un-clipped parts. Second, the POCS method’s accuracy is high even when a large number of samples are clipped. Third, the POCS method is not dependent on the data saturation type, since the main focus is on identifying which samples are clipped. For waveform restoration, the user need only identify and mark the index of the clipped samples; fortunately, the identification of clipped waveform is very easy.

The seismic surface waves have high consistency between early and later arrival parts, which makes it easy to restore the clipped waveforms (i.e., see Fig. 4). In contrast, random phase or impulsive transient signals do not have *a priori* known temporal amplitude characteristics, which makes it much more difficult to reconstruct these types of waveforms. What we are relying on in this work is that our signals meet the basic requirements of our method: the clipped parts share the same frequency content as the non-clipped parts of the waveform. The typical artifacts introduced by our method are smoothed peaks that can result if the true peaks have several sub peaks. Fortunately, this limitation is not too problematic for many waveform-based applications.

Given the known characteristics of seismic surface waves, we can assume the clipped samples exist in only a limited time window that has a relatively simple or narrow-band spectrum. If, however, the spectrum of the seismic signal is complex, our POCS method would produce results with relatively large errors. In these cases, when there are a large number of consecutive clipped samples, when using the POCS method it is important to carefully select the time window so that the instant spectrum within the window does not have a strong variation. There are other situations when our POCS method would introduce strong artifacts such as when the beginning and the ending of the clipped samples time window have different frequency components.

In summary, the POCS restoration method can successfully recover weakly and moderately clipped waveform data, but is not able to process severely clipped data. This restoration process can significantly increase the amount of useable data, and reduce the waste of clipped waveforms. The refinement of large magnitude earthquake catalogues^22^,^23^ and the restoration of clipped waveforms will be helpful in future studies to help us gain a better understanding of seismicity as well as earth’s inner structures, since these restored waveforms can significantly increase the total number of events and stations available for high accuracy waveform-based applications.

We estimate the total number of clipped waveforms that we can restore globally is ~30,000/year. This is a substantial amount of data, providing many more near-field records and a much better azimuthal distribution of stations. These data can help contribute to studies of amplitude-dependencies (e.g. full waveform inversion, focal mechanisms, and receiver function) and in turn produce more robust results. Restored waveforms are particularly important for regions that have sparse station coverage, where restoring waveforms from one or two stations can be essential to determine the source parameters for both earthquake early warning and rapid earthquake response^24^. The restored waveforms would save additional several seconds on determining the earthquake magnitude, because more crucial stations closer to the epicenter would be available in real time after using the proposed method. Besides, both the seismic waveforms obtained by the Apollo^25^ and the electromagnetic waveforms obtained by the lunar rover Yutu are strongly clipped^26^, and the waveform restoration would be helpful to rescue part of these precious data. The source code of waveform restoration using the POCS can be obtained by request from the authors (geophysics.zhang@gmail.com or zhaox@seis.ac.cn).

## Additional Information

**How to cite this article**: Zhang, J. *et al*. Restoration of clipped seismic waveforms using projection onto convex sets method. *Sci. Rep.* **6**, 39056; doi: 10.1038/srep39056 (2016).

**Publisher's note:** Springer Nature remains neutral with regard to jurisdictional claims in published maps and institutional affiliations.

## Supplementary Material

Supplementary Information

## Figures and Tables

**Table 1:** Seismic stations that contain clipped waveform data from the 2013 Mw 6.6 Lushan earthquake in China.

No.	Name	WE	SN	Z
1	BAX	○	○	○
2	MDS	○	○	○
3	TQU	●	●	●
4	YZP	●	●	●
5	GZA	●	●	●
6	EMS	●	●	●
7	XJI	●	●	●
8	CD2	●	●	●
9	JYA	●	●	●
10	YGD	●	●	●
11	SMI	●	●	●
12	WCH	●	●	
13	MBI	●	●	●
14	HMS	●	●	●
15	MXI	●	●	
16	JJS	●		●
17	HSH	●		
18	MEK	●		●
19	MGU	●	●	
20	YJI		●	
21	LBO	●	●	●
22	MNI	●	●	●
23	ZJG	●	●	●
24	HWS	●	●	●
25	XSB	●	●	●
26	BYD	●	●	●
27	YAJ	●	●	●
28	YYC	●	●	●
29	JLI		●	●
30	LZH			●
31	PGE	●	●	●
32	XCO	●	●	●
33	YYU	●	●	●
34	JMG	●		
35	QIJ		●	
36	SMK	●		
37	HLI	●		
38	BZH		●	●

^*^All stations are listed with respect to their epicenter distance, similar to Fig. 6. A blank cell indicates that the waveform for that component is not clipped and does not need waveform restoration. The marker“●” indicates that the waveform is weakly or moderately clipped and can likely be restored using the POSC method. The marker “○” indicates that the waveform is strongly clipped and could not be restored using the POCS method. The three-component waveforms are shown in Fig. 6 and Supplementary Figs 1–2. Seismic stations in WE-component are plotted in Fig. 7.

**Table 2:** The number of clipped and restored waveforms that recorded the 2013 Mw 6.6 Lushan earthquake.

Waveforms	WE	SN	Z	Total	Proportion
Clipped	33	31	29	93	—
Restored	31	29	27	87	93.5%
Failed	2	2	2	6	6.5%

Seismic stations are listed in Fig. 7 and Table 1. The three-component broadband seismic records are shown in Fig. 6 and Supplementary Figs 1–2.
