# Supplementary material for: Restoration of clipped seismic waveforms using projection onto convex sets method
Source: Sci Rep. 2016 Dec 14;6:39056. doi: 10.1038/srep39056 (PMC5155270; doi:10.1038/srep39056)
Supplement: Supplementary Information [file srep39056-s1.pdf]

# Restoration of clipped seismic waveforms using projection onto convex sets method

**Jinhai Zhang[1], Jinlai Hao[1], Xu Zhao[1*], Shuqin Wang[2], Lianfeng Zhao[1], Weimin Wang[3], Zhenxing Yao[1]**

[1] Key Laboratory of Earth and Planetary Physics, Institute of Geology and Geophysics, Chinese Academy of Sciences, Beijing 100029, China.

[2] School of Information Engineering, Minzu University of China, Beijing 100081, China.

[3] Key Laboratory of Continental Collision and Plateau Uplift, Institute of Tibetan Plateau Research, Chinese Academy of Sciences, Beijing 100101, China

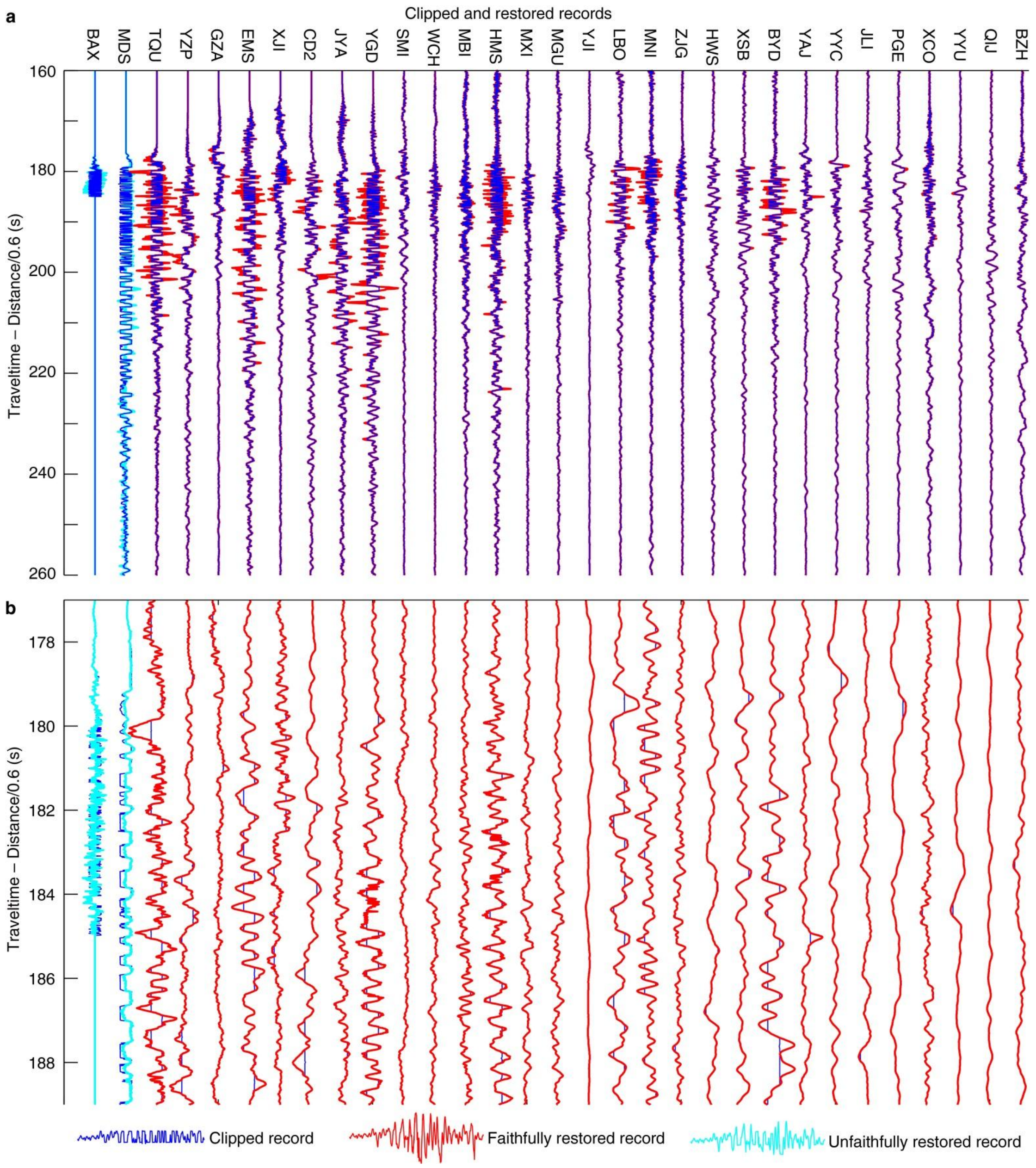


**Supplementary Figure S1. All clipped and restored broadband SN-component records of the 2013 Mw 6.6 Lushan earthquake.** The total number of clipped SN-component records is 31 and we faithfully restored 29 (Table 2). (**a**) Comparison between the clipped (blue) and restored (red) records; (**b**) Zoom in of (**a**) for the time period 177-189. The total number of clipped WE-component records is 33, of which we are able to restored 31 (Table 2). Records that we are not able to restore are shown in cyan. Stations are ordered by epicenteral distance. The records are aligned to show all waveforms within a small time window.

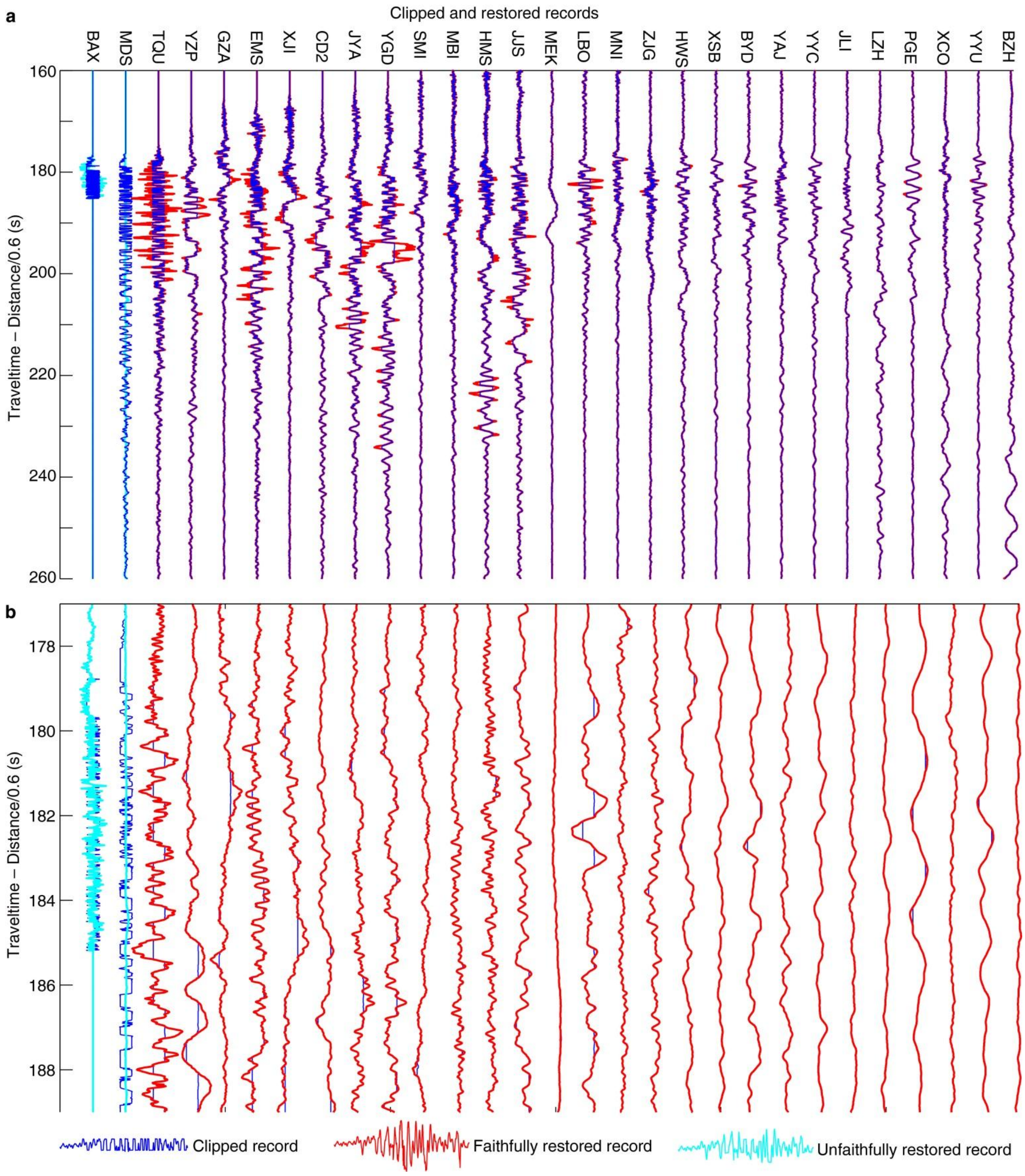


**Supplementary Figure S2. All clipped and restored broadband Z-component records of the 2013 Mw 6.6 Lushan earthquake.** The total number of clipped Z-component records is 29 and we faithfully restored 27 (Table 2). (**a**) Comparison between the clipped (blue) and restored (red) records; (**b**) Zoom in of (**a**) for the time period 177-189. The total number of clipped WE-component records is 33, of which we are able to restored 31 (Table 2). Records that we are not able to restore are shown in cyan. Stations are ordered by epicenteral distance. The records are aligned to show all waveforms within a small time window.
